# Specificity Analysis of Alternative Splicing During *Botrytis cinerea* Infection of Different Hosts

**DOI:** 10.3390/microorganisms14092086

**Published:** 2026-09-17

**Authors:** Siyuan Wang, Panpan Huang, Ping Lu

**Affiliations:** 1 The Key Laboratory for Biology of Crop Pathogens and Insect Pests and Their Ecological Regulation of Zhejiang Province, College of Advanced Agricultural Sciences, Zhejiang A&F University, Hangzhou 311300, China; 202301140210@stu.zafu.edu.cn; 2 College of Agricultural, Yangzhou University, Yangzhou 225009, China

**Keywords:** *Botrytis cinerea*, alternative splicing, different hosts, conservation and specificity

## Abstract

Alternative splicing (AS) is a significant post-transcriptional regulatory mechanism that facilitates environmental adaptation in fungi. However, the conservation of this mechanism and its host specificity during the infection of various hosts by *B. cinerea* remain ambiguous. This study utilized strand-specific RNA sequencing to perform a comparative analysis of genome-wide AS patterns during the infection of tomato and strawberry by *B. cinerea*. A comparative analysis identified a total of 1714 AS events and 1130 associated genes across the two hosts, alongside a significant number of host-specific events. Common alternatively spliced genes were predominantly enriched in processes related to gene expression regulation and signal transduction, whereas host-specific genes displayed distinct functional enrichment characteristics and temporal dynamics in their Percent Spliced In (PSI) patterns. Notably, introns linked to AS were generally longer than those associated with constitutive splicing and predominantly featured canonical GT-AG splice boundaries. Furthermore, the magnitude of PSI changes in host-specific events was greater than that observed in common events. This study reveals that *B. cinerea* exhibits both conserved and host-dependent AS regulatory programs, providing new insights into its post-transcriptional regulatory plasticity during the infection of various plant hosts.

## 1. Introduction

*Botrytis cinerea* is the causative agent of gray mold disease and is classified as a necrotrophic fungal pathogen with a broad distribution and an extensive host range. This pathogen is capable of infecting over 1400 plant species, encompassing a variety of vegetables, fruits, ornamental plants, and economically significant field crops. It can affect multiple plant organs, including leaves, flowers, stems, and fruits, during both pre-harvest and post-harvest stages [1,2]. Due to its broad host range, flexible and diverse infection strategies, high genetic diversity, and strong capacity to develop resistance to fungicides, *B. cinerea* is regarded as one of the most destructive plant pathogenic fungi globally and ranks second among the ten most significant fungal pathogens in molecular plant pathology [3]. During the infection process, *B. cinerea* induces host cell death by simultaneously secreting plant cell wall-degrading enzymes, phytotoxic secondary metabolites, secreted proteins, and other virulence-related factors. This pathogen employs reactive oxygen species-related mechanisms to facilitate its invasion, ultimately acquiring nutrients from the compromised tissues [1]. Tomato (*Solanum lycopersicum*) and strawberry (*Fragaria vesca*) are both significant economic hosts for *B. cinerea*. However, there are notable differences between these two plants regarding tissue structure, cell wall composition, secondary metabolites, nutrient composition, and immune responses [4,5,6,7,8,9,10]. Consequently, the successful colonization of *B. cinerea* on these hosts may depend on both conserved infection mechanisms and regulatory strategies that can adapt to varying host environments.

Alternative splicing (AS) is a significant post-transcriptional regulatory mechanism that enables the processing of the same precursor messenger RNA into multiple distinct mature transcript isoforms. The primary types of AS include intron retention, alternative 5′ splice sites, alternative 3′ splice sites, and exon skipping. Various AS events can modify coding sequences, protein domains, subcellular localization signals, transcript stability, or translation efficiency, thereby enhancing the diversity of the transcriptome and proteome without necessitating an increase in the number of genes [11]. Some alternatively spliced transcripts may contain premature termination codons, which are subsequently cleared through the nonsense-mediated mRNA decay pathway. This indicates that AS not only generates different protein isoforms but also regulates gene expression levels. Historically, introns in fungal genomes were considered fewer and shorter than those in animals and plants, leading to the belief that AS was uncommon in fungi. However, comparative transcriptomic studies have demonstrated that AS is widespread across various fungal groups. Among these, intron retention is the most prevalent type of AS in fungi. Furthermore, the frequency of AS in fungi is associated with multicellular complexity, environmental adaptability, and pathogenicity [12]. During the infection process, fungal pathogens encounter rapid fluctuations in environmental conditions, including nutrient availability, host-derived antimicrobial compounds, oxidative stress, pH levels, and host immune pressure. Consequently, the dynamic regulation of transcript isoform composition and usage ratios may serve as an effective mechanism for pathogenic fungi to adapt to various infection environments.

A growing body of research indicates that AS plays a significant role in the growth, development, and pathogenicity of plant pathogenic fungi. In the rice blast fungus *Magnaporthe oryzae*, genome-wide analyses have revealed extensive changes in transcript isoforms between the vegetative growth stage and various stages of host infection [13]. Multiple genes encoding small secreted proteins, including the effector-related gene *BAS3*, can produce various AS transcripts. This suggests that AS may play a role in the fungal infection process and in the regulation of effector composition [14]. Further research on the necrotrophic fungus *Sclerotinia sclerotiorum*, which is closely related to *B. cinerea*, demonstrates that its AS patterns undergo extensive reprogramming during plant infection. By comparing the transcriptomes of *S. sclerotiorum* under in vitro culture conditions and during the infection of six different host plants, researchers identified multiple AS genes encoding predicted secreted proteins [15]. They found that the relative abundance of certain transcript isoforms varies depending on the host plant. In addition, time-course analysis of the infection process of *Brassica napus* by *S. sclerotiorum* revealed that a significant number of differentially spliced genes did not demonstrate differential expression at the overall transcript level. This finding indicates that AS serves as an independent regulatory layer that is not fully captured by conventional differential gene expression analyses [16]. In summary, post-transcriptional regulation mediated by AS may play a crucial role in fungal developmental stage transitions, virulence regulation, and adaptation to host-associated environments.

Compared to other plant pathogenic fungi, our understanding of AS in *B. cinerea* remains relatively limited. Our research group previously annotated 129 genes encoding the major components of the spliceosome in *B. cinerea* and identified 7466 AS events derived from 3308 genes. Many spliceosome-related genes exhibited elevated expression levels during the early stages of infection, particularly at 12 and 24 h post-inoculation. Furthermore, most of the identified AS events demonstrated clear specificity related to the infection stages. These findings confirm that extensive and dynamic AS occurs during the host colonization by *B. cinerea* [17]. However, this study analyzed only a single host, the tomato, which limits our ability to ascertain whether the observed AS events represent conserved responses common to infections across different hosts or regulatory patterns induced by specific host environments.

This study conducts a transcriptome-wide comparative analysis of AS during the infection of tomato and strawberry by *B. cinerea*. We systematically identify and classify different types of AS events, distinguishing between commonly expressed and host-specific alternatively spliced genes and events in the two hosts. Additionally, we compare their temporal dynamic patterns and splicing levels across different hosts. Furthermore, through functional enrichment analysis, we explore the biological processes involved in conserved and host-dependent AS, while analyzing the positions, lengths, splice site characteristics, and splicing efficiencies of relevant introns within genes. By combining the presence of AS events with quantitative changes in transcript isoform usage ratios, this study aims to identify which splicing characteristics of *B. cinerea* are conserved during plant infection and which are influenced by the host environment. The findings reveal the post-transcriptional regulatory plasticity of *B. cinerea* and provide a theoretical basis for understanding how AS facilitates the colonization of plant hosts with different phylogenetic relationships and physiological characteristics by a broad-host-range phytopathogenic fungus.

## 2. Materials and Methods

### 2.1. Plant, Fungal Pathogen and Inoculations

The strawberry plant used in this study is *Fragaria vesca*. This study used the wild-type strain B05.10 [18] of *B. cinerea* for infection experiments. Conidia were harvested from the culture and washed with sterile water, and the final concentration was adjusted to 5 × 10^6^ conidia/mL to prepare an inoculum suspension for inoculating strawberry leaves. The leaves of the seedlings were immersed in the conidia suspension for 3 min, and leaf samples were collected at 0, 2, 4, 8, and 12 h, respectively. Three replicate experiments (each replicate containing six seedlings) were conducted during the infection stage. All samples were frozen in liquid nitrogen and stored at −80 °C.

### 2.2. RNA Library Construction and Strand-Specific Sequencing

Total RNA was extracted using the RNA prep Pure Plant Kit (TIANGEN Biotech (Beijing) Company Limited, Beijing, China). RNA samples with a RIN value >= 8 were selected for strand-specific RNA seq. For the subsequent process, strand-specific complementary DNA (cDNA) synthesis was carried out utilizing the NEBNext^®^ Ultra™ DNA Library Prep Kit (New England Biolabs, Incorporated, Ipswich, MA, USA). Following the synthesis, sequencing of the cDNA was conducted on the Illumina HiSeq^®^ 2500 platform (Illumina, Incorporated, San Diego, CA, USA), utilizing a paired-end read mode with a length of 2 × 150 base pairs.

### 2.3. Transcriptome Assembly

Low-quality reads were eliminated and adapter sequences were trimmed from the raw reads through the utilization of NGS QC Toolkit version 2.3.3 (Phred Q20, ≥70% bases) [19]. The cleaned reads were aligned with the reference genome of *B. cinerea* (ASM83294v1, Ensembl fungi v53) via HISAT2 version 2.04 (--dta-cufflinks, --rna-strandness RF) [20]. Using StringTie version 2.1.3 [21], the transcriptome was compiled with de novo annotation. The expression values were represented as transcripts per kilobase million (TPM) using Salmon v1.9 (-l ISR --validateMappings) [22]. Any transcript showing an expression value of less than 1 TPM across all samples was categorized as predicted but not detected. Only the transcripts identified as detected were used for further analysis.

### 2.4. Identification of Alternative Splicing

The landscapes of AS were generated through the use of SUPPA v2.2.1 (--MergePval A --StrandSpecific R --SpliceCons TRUE) [23]. For each AS event, the percent splicing index (PSI) was determined using SUPPA2 v2.2.1. To compute the TPM for transcripts across each sample, Salmon v1.9 was utilized.

## 3. Results

### 3.1. Identification of Alternative Splicing Events During B. cinerea Infection of Strawberry Leaves

**Figure 1:**
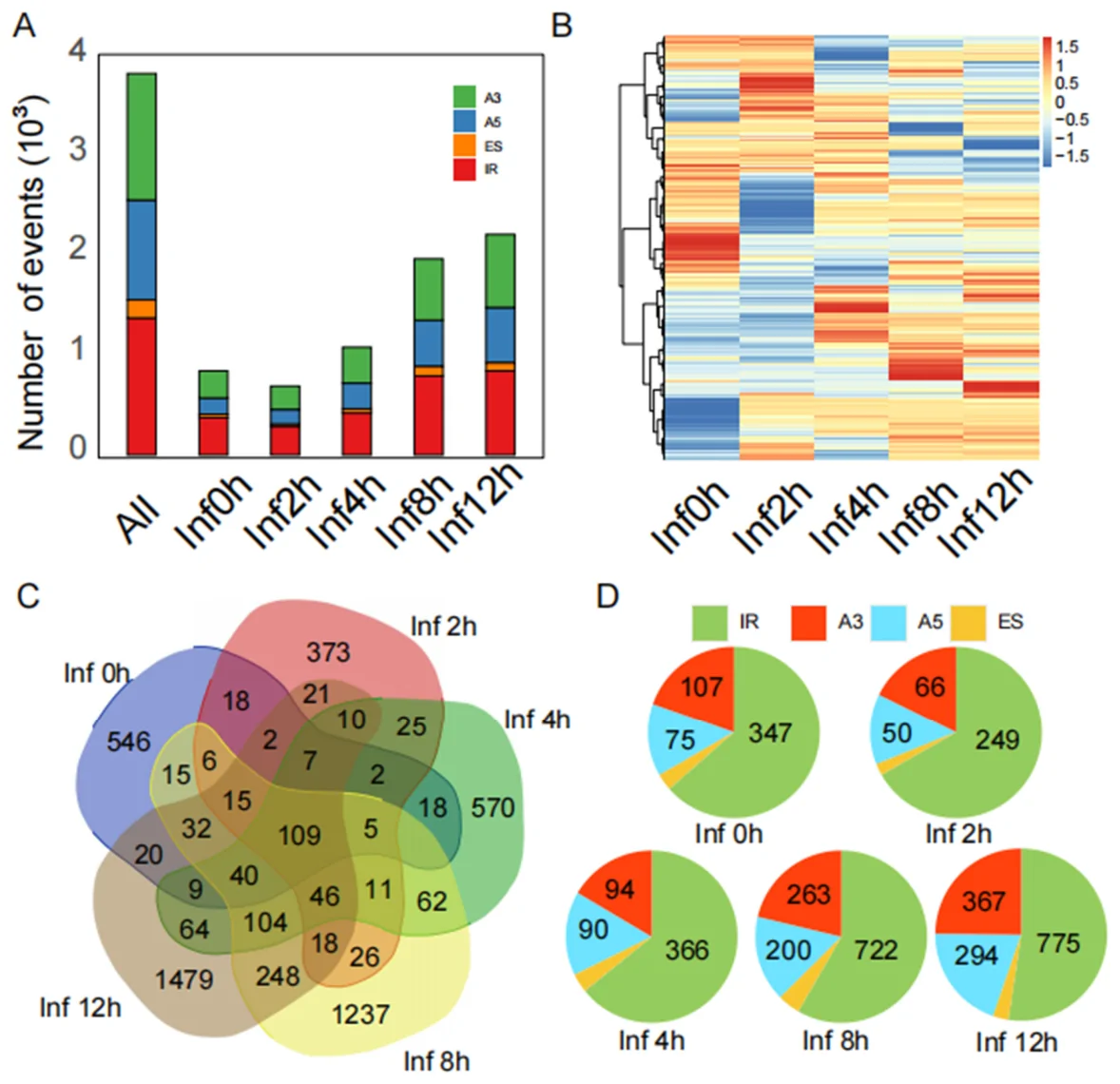
Identification of alternative splicing events during *B. cinerea* infection of strawberry leaves. (**A**) Statistics of the number and types of AS events detected in strawberry leaves infected by *B. cinerea* during 0, 2, 4, 8, and 12 h. “All” represents the total number of non-redundant AS events identified across all infection time points. IR, intron retention; A5, alternative 5′ splice site; A3, alternative 3′ splice site; ES, exon skipping. (**B**) Heatmap of Percent Spliced In (PSI) values for AS events across different infection stages. Colors represent normalized PSI values, with red indicating higher splicing levels and blue indicating lower splicing levels. (**C**) Common and stage-specific distribution of AS events at different infection time points of 0, 2, 4, 8, and 12 h. Numbers in each sector indicate AS events specific to a particular time point or shared among multiple time points. (**D**) Type composition and quantitative statistics of specific AS events at different infection stages.

During the infection of strawberry by *B. cinerea*, a total of 3843 AS events (Table S1) were identified in 2160 genes. These AS events were classified into four basic types for analysis: intron retention (IR), alternative splice donor (A5), alternative splice acceptor (A3), and exon skipping (ES). Across all sequencing time points, IR was the most predominant AS type, followed by A3 and A5, while ES was the least abundant (Figure 1A). The splicing percentage of each AS event varies at different time points, with a large number of active splicing events occurring in the early infection stage (0 h, 2 h) and relatively fewer from 4 h to 12 h post-infection (Figure 1B). There are a large number of stage-specific AS events at different stages of infection, with a large number of specific AS events at 8 h post-infection (1237) and 12 h post-infection (1479) (Figure 1C). Among these stage-specific AS events, intron retention (IR) was the predominant type, followed by A3 splice sites and A5 splice sites, with ES events being the least numerous (Figure 1D).

### 3.2. Comparison of Alternative Splicing Events During B. cinerea Infection of Strawberry and Tomato Leaves

In the early stages of this study, we systematically identified AS events expressed in tomato leaves during infection by *B. cinerea* [17]. Subsequently, we compared the similarities and differences between AS expressed in strawberry leaves during infection by *B. cinerea* and those expressed in tomato.

**Figure 2:**
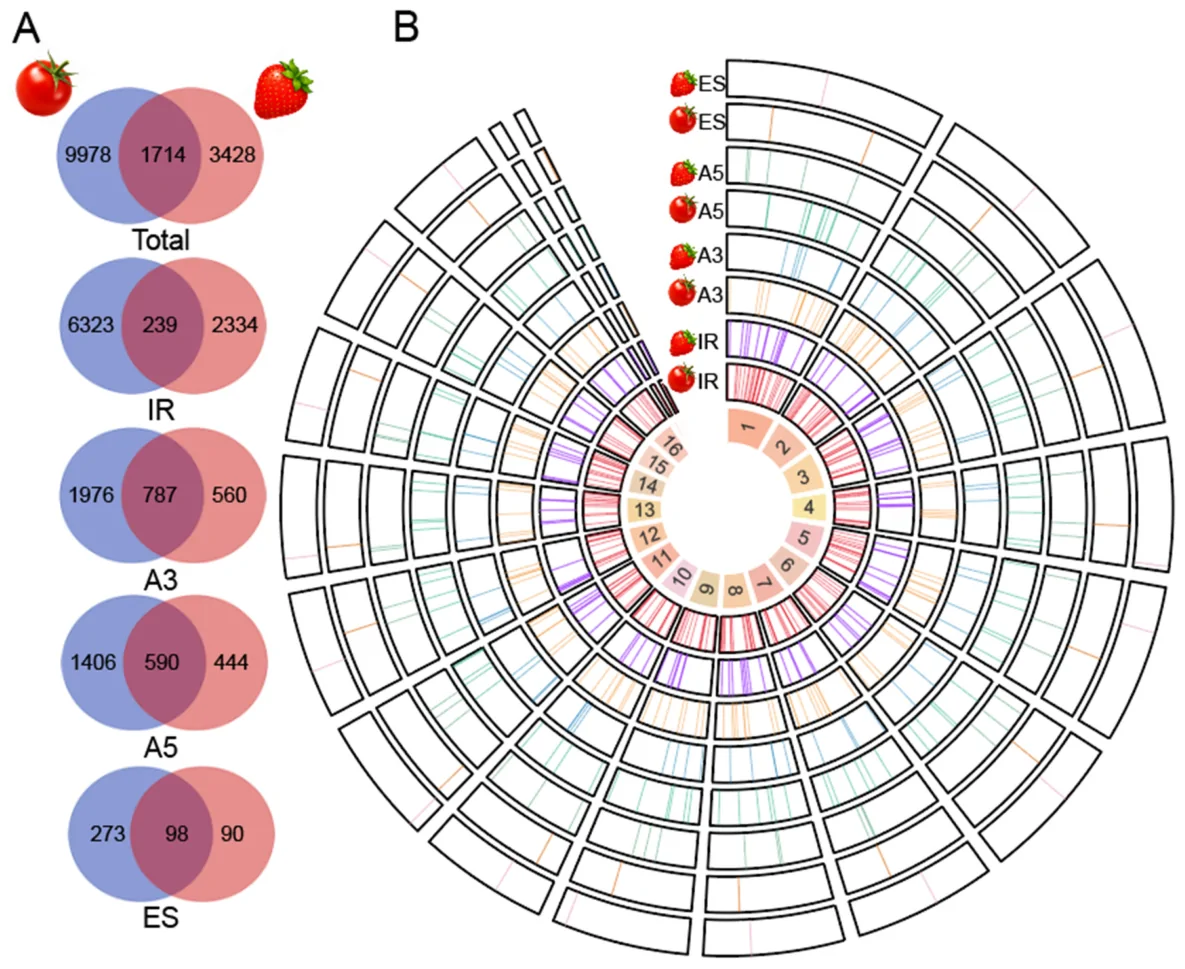
Similarities and differences in alternative splicing events and chromosomal distribution during the infection of strawberry and tomato by *B. cinerea*. (**A**) Statistics of commonality and host-specificity of AS events detected during *B. cinerea* infection of strawberry and tomato leaves. (**B**) Distribution of four types of AS events on the chromosomes of *B. cinerea* during the infection stages of strawberry and tomato.

Initially, we examined the relationship between the two entities based on their genomic positions. During the infection process, we identified a total of 1714 co-expressed AS events (Figure 2A). Among these co-expressed events, the proportion of IR types was relatively low, whereas the A5, A3, and ES types constituted a significantly higher proportion (Figure 2A). We analyzed the chromosomal distribution of four types of AS events in two plant species to investigate whether there is a positional preference specific to each species. The results indicated that the four types of AS events were almost evenly distributed across the 18 chromosomes in both species, with no observed specificity related to either species (Figure 2B). Such AS response characteristics exhibit host specificity, indicating that distinct plants trigger differential AS events when infected by the same phytopathogen, which contributes to diverse plant–pathogen interaction regulatory mechanisms.

### 3.3. Analysis of Co-Expressed Alternatively Spliced Genes in Tomato and Strawberry Infected by B. cinerea

**Figure 3:**
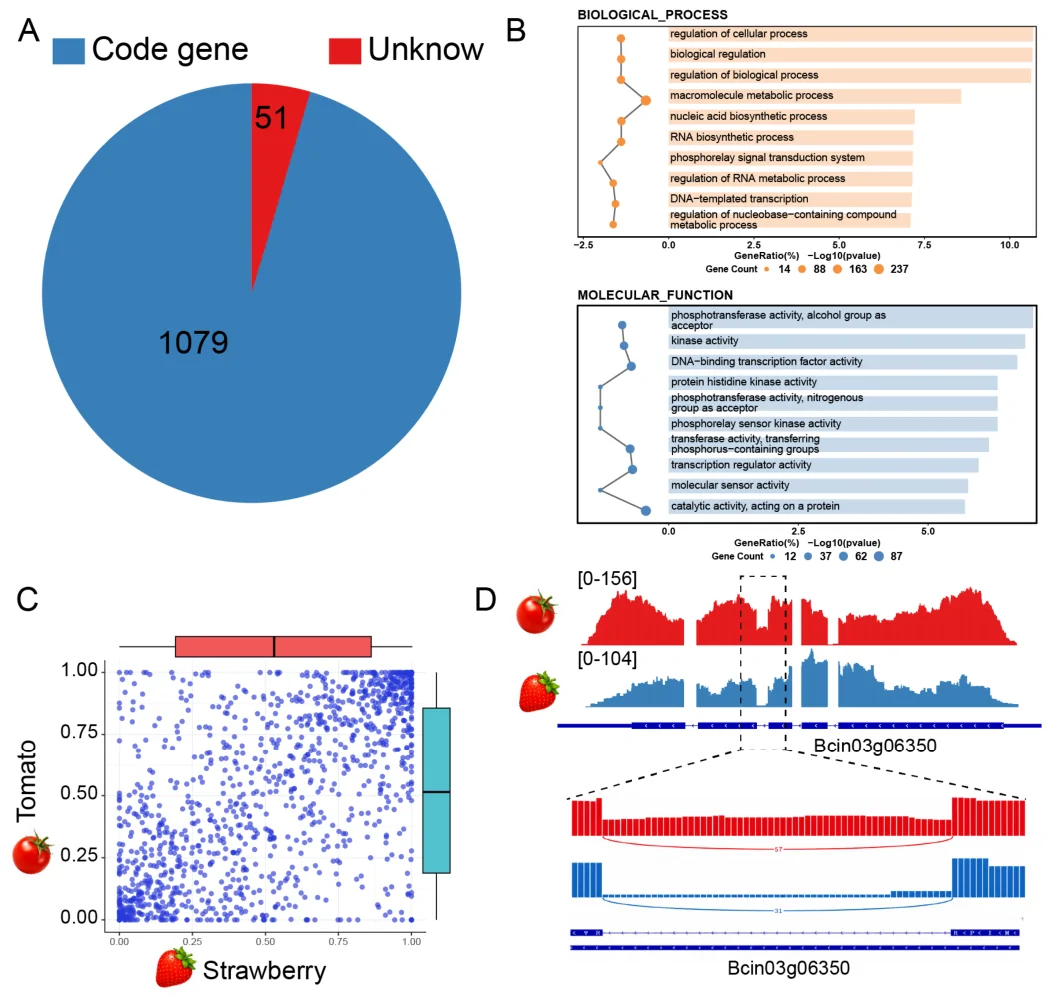
Characterization of alternative splicing events commonly detected during *B. cinerea* infection of strawberry and tomato. (**A**) Overlap statistics of genes corresponding to common AS events with existing genome annotations of *B. cinerea*. (**B**) Co-expression functional enrichment analysis of genes related to AS events. (**C**) Correlation analysis of Percent Spliced In (PSI) values for common AS events at 12 h post-infection of strawberry and tomato leaves by *B. cinerea*. Each scatter point represents an AS event detected during infection of both hosts, with the x-axis and y-axis indicating the PSI value of the event at 12 h post-infection of strawberry and tomato, respectively; the box plots above and to the right show the overall distribution of PSI values in the two hosts. (**D**) RNA-seq read coverage and splicing junctions of *B. cinerea* infecting strawberry and tomato leaves at 12 h. Red and blue indicate samples infecting tomato and strawberry, respectively; arc connections represent exon-spanning splicing reads; dashed boxes mark regions where splicing ratios differ between the two host infection conditions.

To investigate the functions of alternatively spliced genes co-expressed during infection of tomato and strawberry, we conducted a comprehensive secondary analysis. Among the 1130 genes that exhibited AS events during both tomato and strawberry infection stages, 95.5% have been annotated as coding genes, and only 51 genes are newly identified (Figure 3A).

To analyze the potential functions of commonly expressed AS genes in strawberry and tomato during *B. cinerea* infection, we performed Gene Ontology (GO) enrichment analysis. The results identified 52 significantly over-represented GO terms (FDR < 0.05), comprising 38 biological process (BP) terms and 14 molecular function (MF) terms, with no significantly enriched cellular component (CC) terms identified (Table S2). In the BP category, co-expressed alternatively spliced genes were mainly enriched in regulation of cellular process, regulation of biological process, and biological regulation, with these terms showing the highest enrichment significance. Additionally, macromolecule metabolic processes were also significantly enriched (Figure 3B). These results indicate that co-expressed alternatively spliced genes in strawberry and tomato are extensively involved in regulating gene expression at both the transcriptional and post-transcriptional levels. BP terms related to signal perception and transmission are also significantly enriched, including the phosphorelay signal transduction system, cell communication, signal transduction, intracellular signal transduction, and phosphorylation. Consistently, the MF categories of kinase activity, protein kinase activity, protein histidine kinase activity, phosphorelay sensor kinase activity, and transferase activity that transfers phosphorus-containing groups are significantly enriched.

Although the same AS event of a given gene occurs during infection in both strawberry and tomato, the splicing ratios may differ. To further investigate this, we compared the splicing index statistics of AS events for the same gene at 12 h post-infection. The results revealed that the Percent Spliced In (PSI) values of these co-occurring events were widely distributed across the two species. Some events clustered in the lower-left and upper-right corners, indicating similar splicing ratios in strawberry and tomato, which suggests the potential presence of conserved splicing regulatory patterns. However, a significant number of events deviated from the diagonal line, indicating that while these events were detected in both species, their splicing ratios differed markedly, suggesting that common AS events in response to *B. cinerea* infection may exhibit species-specific splicing regulatory features (Figure 3C). The gene Bcin03g06350 demonstrates AS within the third intron during the infection of both strawberry and tomato. However, the intron retention rate is significantly higher during tomato infection compared to strawberry infection (Figure 3D).

### 3.4. Analysis of Specifically Expressed Alternative Splicing Genes in Tomato and Strawberry Infected by B. cinerea

**Figure 4:**
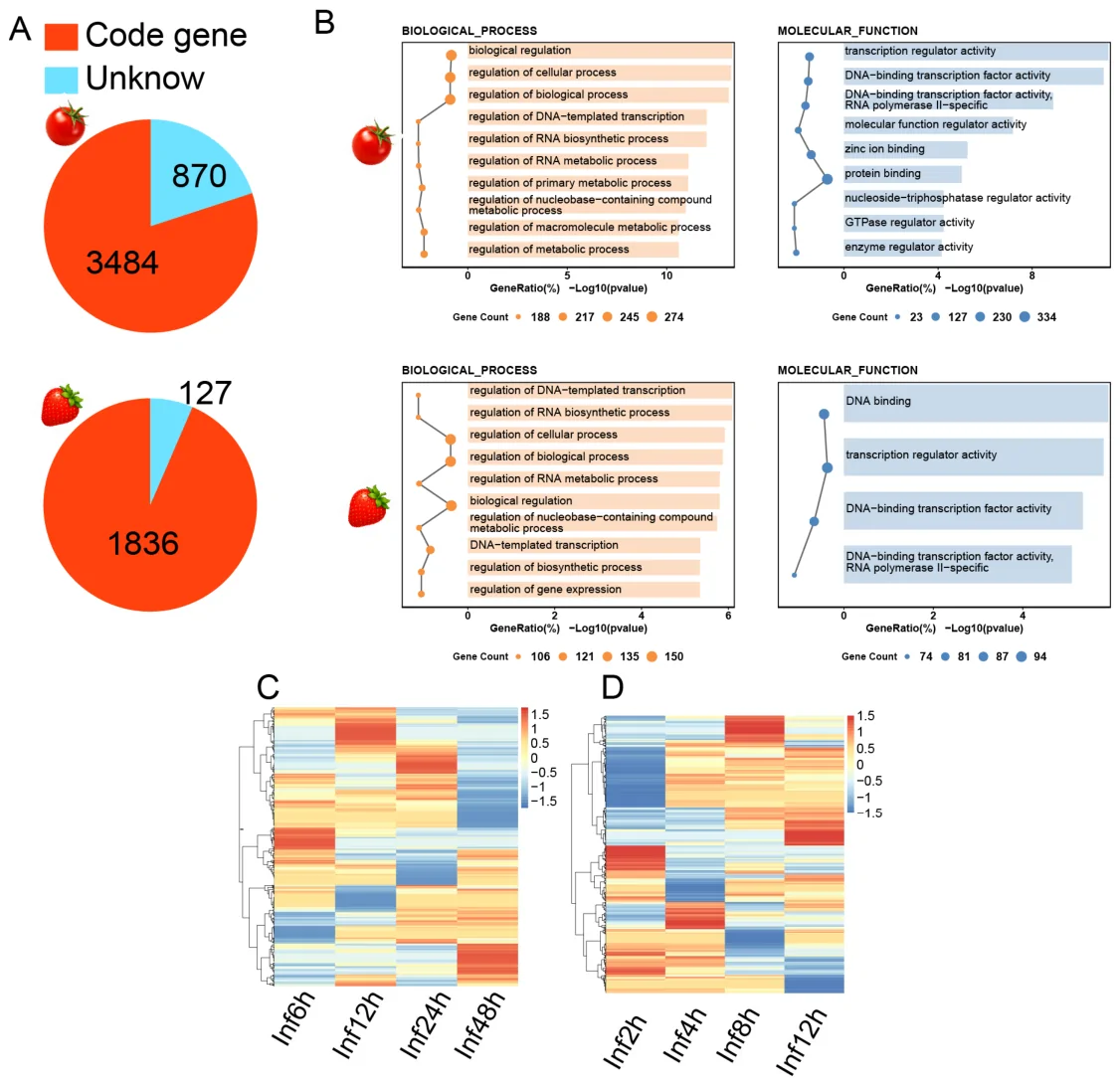
Gene characteristic analysis of host-specific alternative splicing events during infection of tomato and strawberry by *B. cinerea*. (**A**) Comparison of the genes belonging to host-specific AS events during infection of tomato and strawberry with the existing genome annotation of *B. cinerea*. (**B**) Gene Ontology (GO) functional enrichment analysis of genes associated with specific AS events during the infection process of tomato and strawberry. (**C**,**D**) Heatmap analysis of percent spliced in index of AS events specific to *B. cinerea* infection in tomato and strawberry, respectively.

To analyze the potential functions of specifically expressed AS genes in strawberry and tomato during *B. cinerea* infection, we conducted a thorough analysis. During the infection of tomato leaves by *B. cinerea*, we identified 4354 genes associated with specifically expressed AS events, of which 3484 (80.0%) were annotated as coding genes. In contrast, during the infection of strawberry leaves, we found 1963 genes involved in AS events, with coding genes comprising 93.5% of the total (Figure 4A).

We also conducted a functional enrichment analysis on the genes associated with AS events that were specifically expressed during the infection of tomato and strawberry. The analysis revealed that the tomato infection stage exhibited a greater number of enriched GO terms for specifically expressed genes, totaling 41 significant GO terms (Table S3), in contrast to the 23 significant GO terms identified for the strawberry infection stage (Table S4). The GO enrichment analysis during tomato infection revealed functions that are either unique to or more prominent in tomato, such as signal transduction, cell communication, intracellular signaling, response to stimuli, and cellular responses to stimuli (Table S3). This finding indicates that when *B. cinerea* infects tomato, the specifically expressed genes are involved in a broader spectrum of functions with more-complex regulatory mechanisms (Figure 4B). In contrast, during the infection of strawberry, the GO terms enriched by genes associated with AS events include DNA binding, transcription regulator activity, DNA-binding transcription factor activity, RNA polymerase II specificity, regulation of DNA-templated transcription, and regulation of the RNA biosynthetic process (Table S4). Although the infection stage of strawberry also had obvious transcriptional regulatory enrichment, compared with tomato, there were fewer specifically enriched terms such as signal transduction, response to stimulus, and protein modification (Figure 4B).

Additionally, we calculated the splicing index for stage-specific expressed AS events in both infected tomato and strawberry. The AS events specific to infected tomato exhibited a slightly higher PSI around Inf 12 h; however, there was an overall decrease by Inf 48 h. Many events reached elevated levels at Inf 6 h or Inf 12 h, while another subset demonstrated significant increases or decreases at Inf 48 h. Notably, the differences observed at Inf 48 h compared to earlier time points were more pronounced, indicating a significant transition in splicing states during the late infection stage (Figure 4C). Infection-specific AS events in strawberry exhibited clear stratification at four time points (2 h, 4 h, 8 h, and 12 h). Many events showed relatively low PSI at 2 h but relatively high PSI at 8 h or 12 h, indicating a significant shift in splicing ratios during the middle and late stages of infection (Figure 4D).

### 3.5. Analysis of Intron Features of Common and Specific Alternative Splicing Events in B. cinerea-Infected Tomato and Strawberry

Introns and their flanking splice sites constitute the fundamental structural basis for pre-mRNA splicing. The selective retention of introns or the recognition of different splice sites can lead to various forms of AS. Factors such as intron length, genomic location, and the sequence characteristics of splice sites may all influence the spliceosome’s recognition efficiency of these sites, thereby regulating the formation of distinct transcripts. Consequently, analyzing the structural and sequence features of introns involved in AS is crucial for elucidating the potential regulatory mechanisms of AS in strawberry and tomato during infection by *B. cinerea*.

**Figure 5:**
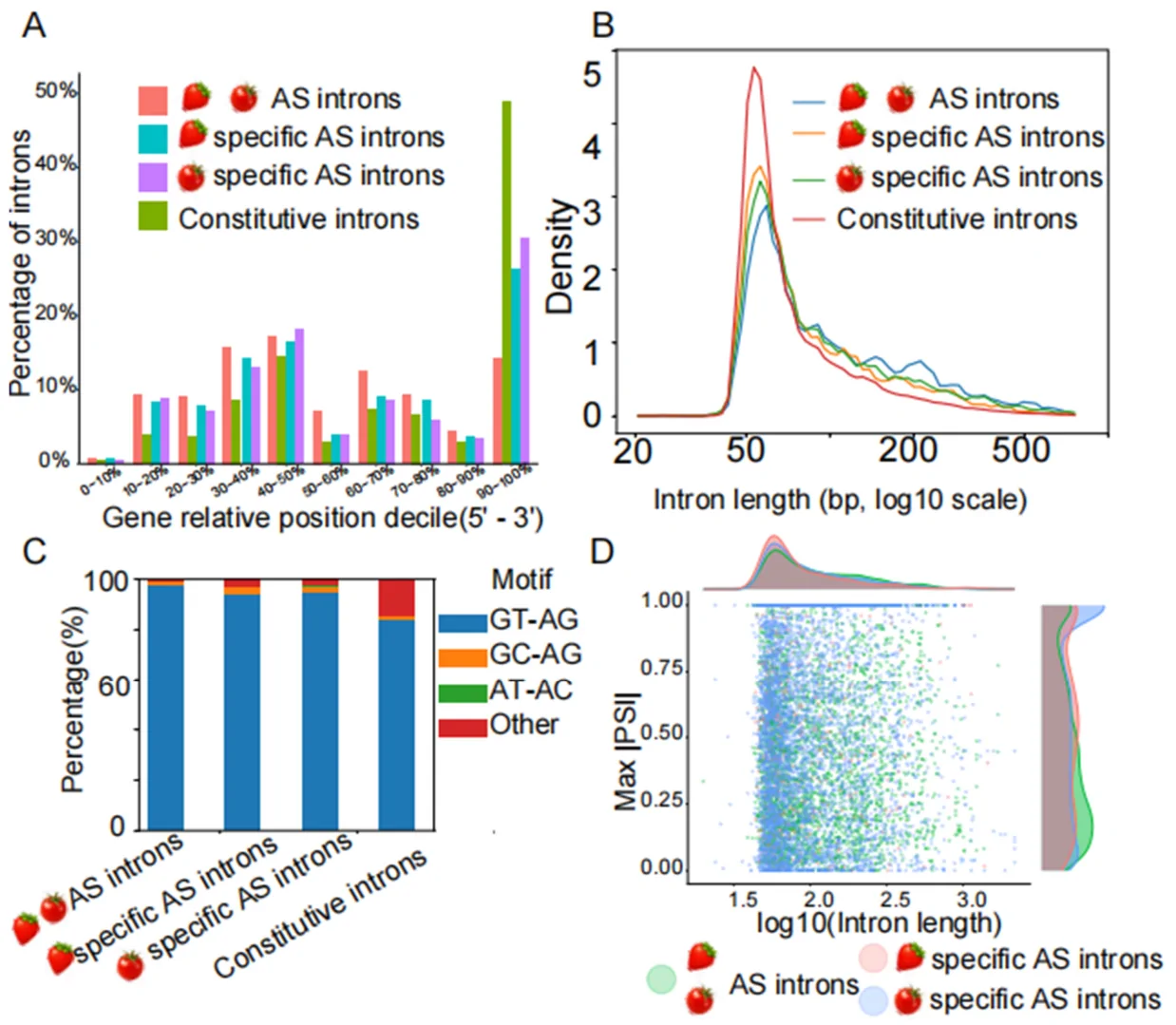
Characteristic statistics of different types of alternatively spliced introns in *B. cinerea*. (**A**) Distribution of different intron types according to normalized intron ordinal position, including AS introns co-detected in strawberry and tomato, strawberry-specific AS introns, tomato-specific AS introns, and constitutive introns. (**B**) Statistics of length distribution of different types of introns. (**C**) Statistics of sequence preference at splice sites of different types of introns. (**D**) The relationship between the lengths of three types of alternatively spliced introns and the PSI index.

We categorized introns into four primary types: co-expressed introns in tomato and strawberry, specifically expressed introns in tomato, specifically expressed introns in strawberry, and constitutive introns. Initially, we examined the positional distribution of introns within their corresponding coding genes. Co-expressed alternatively spliced (AS) introns were not significantly concentrated at the first intron, comprising only 22.0% of the total. In contrast, the proportion of specifically expressed AS events in strawberry at the first intron was 30.7%, while for tomato, it was 43.6%. Constitutive introns exhibited a higher prevalence at the first intron, reaching 45.6%. Overall, alternatively spliced introns appear to be more concentrated in the proximal regions of the gene compared to constitutive introns, which tend to be more frequently located in relatively distal regions (Figure 5A).

The length distributions of alternatively spliced (AS) introns differ significantly from those of constitutive introns. Constitutive introns are predominantly concentrated in the short intron range, exhibiting a median length of 61 bp. In contrast, the median lengths of co-expressed, strawberry-specific, and tomato-specific AS introns are 82 bp, 68 bp, and 72 bp, respectively. Length interval analysis reveals that the proportion of introns ≤100 bp is highest among constitutive introns, whereas AS introns show a notable increase in the 101–500 bp range, particularly for co-expressed AS introns, which account for the highest proportions in both the 101–200 bp and 201–500 bp intervals. These findings suggest that introns involved in AS are generally longer and may possess more-complex cis-regulatory sequences or weaker splicing recognition features. Among these, co-expressed AS introns exhibit the longest lengths, indicating that they may represent relatively conserved and structurally complex splicing regulatory sites during the infection of various hosts by *B. cinerea* (Figure 5B).

The analysis of the 5′ and 3′ end boundary sequences of various introns reveals that the three types of AS-related introns are predominantly characterized by canonical GT-AG splice boundaries. Among these, the co-expressed AS introns exhibit the highest proportion of GT-AG at 97.8%, while the strawberry-specific and tomato-specific AS introns show GT-AG proportions of 94.4% and 95.0%, respectively. Furthermore, GC-AG, classified as a minor type, demonstrates a slight increase in host-specific AS introns. Overall, AS-related introns primarily adhere to the canonical GT-AG splicing rules; however, the observed increase in GC-AG and the presence of a few non-canonical boundaries in host-specific AS introns indicate that these may possess weaker or more-flexible splicing recognition characteristics (Figure 5C).

To further analyze the regulatory dynamics of various types of AS events, this study correlated the magnitude of PSI changes with AS types and intron structural features. The results indicated that, compared to co-expressed AS events, host-specific AS events exhibited greater magnitudes of PSI changes. Notably, the IR type in tomato-specific AS events displayed the highest level of dynamic variation, while the A3 type in strawberry-specific AS events demonstrated a strong splicing regulatory response. These findings suggest that different host environments may achieve post-transcriptional adaptation by regulating distinct splicing patterns (Figure 5D).

## 4. Discussion

Currently, there are few studies investigating the identification of AS events during the host infection stage of *B. cinerea*, with the only previously published research being conducted by our group on the infection of tomato by *B. cinerea* [17]. The question of whether differences in AS exist when *B. cinerea* infects different hosts remains to be explored. This study presents a comparative transcriptome analysis of AS during the infection of both strawberry and tomato.

After comparing the infected tomato, we identified a total of 1714 AS events that were common to both hosts, in addition to a greater number of tomato-specific and strawberry-specific AS events. Genes exhibiting common AS were predominantly associated with cellular regulation, signal transduction, phosphorylation, and kinase activity. In contrast, host-specific AS genes displayed distinct patterns of functional enrichment. Furthermore, even when the same AS event was observed in both hosts, the PSI could vary significantly, as illustrated by the intron retention event of the Bcin03g06350. Additional analysis of intron structural features revealed differences among shared, host-specific, and constitutive splicing introns regarding length, splice site composition, and dynamic changes in PSI. These findings suggest that the regulation of AS in *B. cinerea* involves both conserved regulatory components commonly employed during the colonization of different plant hosts and adaptable regulatory components that modulate the proportion of transcript isoforms in response to the host environment.

The 1714 AS events commonly detected in strawberry and tomato may represent a component of the conserved post-transcriptional regulatory program that *B. cinerea* employs when infecting various hosts. Genes associated with these events are significantly enriched in functions such as biological regulation, intracellular signal transduction, phosphorylation, histidine kinase activity, and protein kinase activity. Signaling proteins play a crucial role in sensing host-derived nutrients, oxidative stress, pH changes, antimicrobial metabolites, and other environmental signals. Consequently, by modulating the transcript structures of relevant genes, *B. cinerea* may further adjust the output of signal transduction without altering the overall expression levels of these genes. Functional studies in other plant pathogenic fungi support the biological significance of this regulatory mechanism. In *Fusarium graminearum*, the serine/arginine-rich protein gene *FgSRP1* generates two isoforms via AS, both of which are essential for normal fungal growth and deoxynivalenol synthesis [24]. In *Magnaporthe oryzae*, the intron retention of the *MoPTEN* gene results in the production of two protein isoforms that exhibit distinct biochemical properties. Both isoforms play crucial roles in conidiation, appressorium development, and invasive growth [25]. These experimentally validated studies demonstrate that changes in the proportion of transcript isoforms can directly affect fungal phenotypes. This study demonstrates that the presence of the same AS event in both hosts does not necessarily imply that its quantitative regulation is also conserved. The PSI values of numerous shared AS events significantly deviate from the diagonal line corresponding to completely identical splicing levels between the two hosts. Additionally, the intron retention ratio of the Bcin03g06350 gene exhibits a clear host-dependent pattern. This indicates that the conservation of splice site selection and the conservation of the usage ratio of transcript isoforms should be regarded as two distinct regulatory attributes. In other words, while shared AS events can determine the transcripts a gene can produce, the relative abundance of different transcripts may still vary depending on the specific host environment.

A substantial number of tomato-specific and strawberry-specific AS events further suggest that post-transcriptional regulatory plasticity may play a role in the development of the broad host range of *B. cinerea*. Tomato-specific alternatively spliced genes are primarily enriched in functions such as signal transduction, cell communication, response to stimuli, and protein modification. In contrast, strawberry-specific alternatively spliced genes exhibit a more significant enrichment in functions such as DNA binding, transcription factor activity, and regulation of RNA biosynthetic processes. Although these functional speculations necessitate further experimental validation, the differing enrichment patterns imply that *B. cinerea* does not utilize an identical regulatory program across all compatible hosts. Rather, it may selectively modulate the transcript isoform composition of various functional modules in response to variations in host cell wall composition, nutrient availability, secondary metabolites, redox environment, and immune pressure. Recent studies on the cross-host infection of *B. cinerea* provide further support for this explanation. Genome-wide association analyses conducted on eight eudicot hosts indicate that both general virulence and host-specific virulence of *B. cinerea* are highly polygenic. The associated genes are distributed throughout the core genome rather than being controlled by a limited number of loci that determine host range [15]. Another cross-species transcriptomic study across multiple plants further identified a set of conserved lesion-associated transcriptional programs, as well as multiple highly plastic host-induced gene modules capable of responding to specific host environments [26]. The results of this study suggest that a comparable model of “core regulatory program plus plasticity regulatory program” may be present at the level of AS. The four types of AS did not exhibit significant clustering on the chromosomes of *B. cinerea*, which aligns with the generally observed dispersed distribution pattern of the associated regulatory sites. 

This study is the first to investigate the common and distinct characteristics of AS events in *B. cinerea* during the infection of various hosts. Additionally, it is the first to elucidate the potential existence of a similar “core regulatory program plus plastic regulatory program” model at the AS level when phytopathogenic fungi infect different hosts. This work establishes a foundation for further exploration of whether AS events significantly contribute to the broad host range of *B. cinerea*.

## 5. Conclusions

In summary, our study presents the first comparative analysis of common and differential AS events during the infection of two host stages by *B. cinerea*. While a significant number of AS events were shared between tomato and strawberry infections, numerous host-specific AS events were also identified. This indicates that *B. cinerea* employs both a relatively conserved program of alternative splicing regulation across different host environments and a pronounced host-dependent regulatory mechanism. Furthermore, even when identical AS events were detected in both hosts, their splicing ratios exhibited notable differences. This research provides a foundational basis for further investigations into how AS-mediated post-transcriptional regulation contributes to the adaptation of *B. cinerea* to diverse host environments and the establishment of its broad host range.

## Data Availability

The sequence data supporting the findings of this study for the strawberry infection stage have been deposited in the NCBI Sequence Read Archive (SRA) under the primary accession number PRJNA1503197. The data for the infection stage of tomato were downloaded from PRJNA1056687. During the preparation of this work, the author(s) did not use any generative AI or AI-assisted technologies.

## Supplementary Materials

The following supporting information can be downloaded at: https://www.mdpi.com/article/10.3390/microorganisms14092086/s1, Table S1: Genome-wide list of alternative splicing event information; Table S2: Detailed list of co-expression functional enrichment analysis results for genes associated with AS events; Table S3: Detailed list of Gene Ontology (GO) functional enrichment analysis results for genes associated with specific AS events during tomato infection; Table S4: Detailed list of Gene Ontology (GO) functional enrichment analysis results for genes associated with specific AS events during strawberry infection.
